# Treatment of Obstructive Sleep Apnea in Children: Handling the Unknown with Precision

**DOI:** 10.3390/jcm9030888

**Published:** 2020-03-24

**Authors:** David Gozal, Hui-Leng Tan, Leila Kheirandish-Gozal

**Affiliations:** 1 Department of Child Health and the Child Health Research Institute, University of Missouri School of Medicine, Columbia, MO 65201, USA; gozall@health.missouri.edu; 2 Department of Paediatric Respiratory Medicine, Royal Brompton Hospital, London, UK; hlt20@doctors.org.uk

**Keywords:** sleep disordered breathing, diagnosis, sleep studies, adenotonsillectomy, inflammation, CPAP, orthodontics, obesity

## Abstract

Treatment approaches to pediatric obstructive sleep apnea (OSA) have remarkably evolved over the last two decades. From an a priori assumption that surgical removal of enlarged upper airway lymphadenoid tissues (T&A) was curative in the vast majority of patients as the recommended first-line treatment for pediatric OSA, residual respiratory abnormalities are frequent. Children likely to manifest persistent OSA after T&A include those with severe OSA, obese or older children, those with concurrent asthma or allergic rhinitis, children with predisposing oropharyngeal or maxillomandibular factors, and patients with underlying medical conditions. Furthermore, selection anti-inflammatory therapy or orthodontic interventions may be preferable in milder cases. The treatment options for residual OSA after T&A encompass a large spectrum of approaches, which may be complementary, and clearly require multidisciplinary cooperation. Among these, continuous positive airway pressure (CPAP), combined anti-inflammatory agents, rapid maxillary expansion, and myofunctional therapy are all part of the armamentarium, albeit with currently low-grade evidence supporting their efficacy. In this context, there is urgent need for prospective evidence that will readily identify the correct candidate for a specific intervention, and thus enable some degree of scientifically based precision in the current one approach fits all model of pediatric OSA medical care.

## 1. Adenotonsillectomy (T&A)

There is now little doubt that hypertrophy of upper airway lymphadenoid tissues constitutes the most common factor underlying the presence of obstructive sleep apnea (OSA) in children, a condition that was formally identified as a singular disease only in 1976 by Guilleminault and colleagues [1]. As corollary of such repeatedly confirmed fact, adenotonsillectomy (T&A) has become the initial treatment recommended by the American Academy of Pediatrics (AAP) consensus guidelines for pediatric OSA in 2002 and subsequently in 2012 [2,3], and other guidelines around the world echo such recommendations [4,5,6,7,8,9,10]. In more recent years, and particularly since 2006 when we initially described the relatively high prevalence of residual OSA after T&A [11,12], confirmation and realization that, although the severity of OSA will routinely improve after surgery, it can persist in a significant proportion of patients has definitely settled in [13,14,15,16]. Indeed, persistent OSA after T&A may occur between 13% to 29% among children defined as low-risk patients, while residual OSA may be present in up to 75% in higher-risk groups such as in obese children [17,18,19,20,21,22,23,24,25,26,27,28,29,30,31]. Other risk factors for persistence of OSA after surgical intervention include age >7 years, asthma, nocturnal enuresis, allergic rhinitis, and the severity of OSA prior to T&A [20,31,32,33,34]. Such relatively elevated frequency of residual OSA has prompted exploration of biomarkers such as high sensitivity C-reactive protein to detect those at risk [20]. Notwithstanding, given the high probability for improvements in the severity of OSA following T&A, even among those children with a priori a high risk of persistence, T&A surgery has remained the initial treatment option, and justifiably so, especially if endoscopic or imaging data attest to the presence of enlarged lymphadenoid tissues impinging on the airway patency or diameter.

Similar to the drivers leading to the recognition and importance of timely diagnosis and treatment of OSA, identification and effective treatment of residual OSA aims at preventing the ongoing elevated risk for OSA-associated morbidities. It is now assumed quite universally that despite the absence of randomized controlled trials providing compelling evidence to the effect that the presumptive morbidities of pediatric OSA are reversible, the condition imposes an increased risk for deteriorations in school performance, alterations in cognitive and learning capabilities, changes in endothelial function and in blood pressure control homeostasis, as well as increased probability of dyslipidemias and insulin resistance [35,36,37,38,39,40], all of which are putatively ascribed to activation and propagation of OSA-induced oxidative and inflammatory cascades [41].

Long-term studies on the efficacy of T&A are clearly lacking. In a very small cohort of 12 school-age children who underwent re-evaluation four years after T&A treatment of OSA, two-thirds appeared to have normalized their PSG findings [42]. Similarly, of the 23 pre-school children who underwent PSG evaluations three years after T&A, 61% had normal sleep studies [43]. However, considering that >600,000 T&A are performed annually in the US alone, the absence of long-term outcomes and the uncertainties related to the potentially adverse effects of T&A later in life [44,45,46], there is a clear need for improved tracking of large cohorts to gain insight as to the potential lifelong consequences of T&A.

We should also point out that more recent trends have revealed a progressive shift towards tonsillotomy by ENT surgeons with a corresponding decline in tonsillectomies, as there is some evidence indicating reduced post-surgical complication rates in tonsillotomy, such as bleeding, pain scores and related interventions, and briefer recovery period. A meta-analysis that evaluated tonsillectomy and tonsillotomy for OSA in children examined the findings of 10 studies, and showed that there appeared to be no significant differences in the rates of OSA symptomatic relief, quality of life, or in the changes in immune function after surgery [47]. However, tonsillotomy exhibited a much higher risk (more than 3-fold) of OSA recurrence compared to tonsillectomy. Furthermore, symptom recurrence is frequent in younger children and may require another surgery within two years after tonsillotomy because of tonsillar tissue regrowth in a proportion of cases [48]. The 2012 American Academy of Pediatrics Guidelines suggested that current data were insufficient to recommend one surgical technique over the other [3]. However, children undergoing tonsillotomy should be monitored carefully long-term to ensure that OSA symptoms do not recur, and families should be counseled about the possibility of OSA recurrence secondary to tonsillar regrowth.

## 2. Residual OSA

Outside the risk factors delineated above, there are few, if any, studies that have specifically focused on the pathophysiology of residual OSA after T&A. Improved understanding of such elements and their corresponding contributions to any specific child manifesting PSG-based residual OSA after surgery is of paramount importance to formulate a intervention strategy that is not only efficacious but is precisely aligned to counteract the effects of such risk factors—in other words, personalized medicine. Conceptually, there are three major categories that are implicated in the emergence of residual OSA: (i) anatomical (mal)development; (ii) upper airway tissue deposition or infiltration; and (iii) increased airway collapsibility. The first group is usually prenatally determined and includes issues such as micrognathia, macroglossia, and midface hypoplasia [49]. The second subset of cases will normally develop after birth, and the narrowing of the airway is usually present when obesity or substrate deposition in the upper airways occurs, as illustrated by mucopolysaccharidoses. Increased upper airway collapsibility is commonly the result of local inflammation, e.g., asthma or allergic rhinitis, or is the consequence of perturbations in neural reflexes and dysfunctional recruitment of the upper airway musculature, and is commonly observed among patients with cerebral palsy or those suffering from neuromuscular diseases. In most of the patients with OSA, several contributing factors are normally present in the same patient. In light of the usually multifactorial pathophysiology of pediatric OSA, simply performing T&A is unlikely to be sufficient to “cure” the disease in the majority of patients. 

Obesity is one of the obvious risk factors for persistent OSA, and the current worldwide obesity epidemic, not just in developed but also in developing countries, will likely further increase the proportion of children after T&A who manifest residual disease. It is now well established that obesity per se is a condition manifesting intrinsic low-grade systemic inflammation that poses significant risks for cardiovascular and metabolic disease. Accordingly, it is not surprising that the concurrent presence of obesity in children with OSA may amplify the morbid effects of OSA. As such, identification of any obese children with residual OSA should become a priority to prevent downstream long-term consequences. In phase 2 of the NANOS study, a prospective multicenter study of obese children recruited from primary care clinics in the community [50] showed that obese children with moderate/severe OSA with significant adenotonsillar hypertrophy who underwent T&A had improvements in their OSA severity, but >40% had residual OSA. In an attempt to identify how obesity causally contributes to the risk of residual OSA, Nandalike et al. evaluated by means of MRI scans 27 obese children with OSA (mean age 13.0 ± 2.3 y) before and after T&A [51]. Although volume increases in the nasopharynx and oropharynx were apparent after T&A, substantial amounts of lymphadenoid tissues were still impinging on the airways along with increases in the volume of the soft palate, tongue and head and neck subcutaneous fat.

## 3. Non-Surgical Approaches to Pediatric OSA

### 3.1. Nasal Corticosteroids

The initial study examining the potential benefits of systemic corticosteroids in pediatric OSA yielded negative results [52,53], A subsequent study led by Brouillette and collaborators involved a six-week course of intranasal fluticasone or placebo among 25 children with OSA. In that study, significant improvements in the fluticasone treatment arm emerged, while some degree of worsening of the AHI occurred in the placebo-treated group [11,53]. A subsequent RCT study by our research group relied on the important observation that corticosteroids markedly inhibit adenotonsillar tissue proliferation and inflammation in an in vitro model and corroborated previous findings while expanding their scope to less severely affected patients, thereby expanding the decision tree in pediatric OSA to include anti-inflammatory therapy in lieu of T&A in younger patients with mild OSA [54,55,56]. Other studies have further confirmed the efficacy of intranasal corticosteroids, either alone or in combination with other anti-inflammatory agents [57,58,59].

### 3.2. Montelukast

The initial open-labeled study examining a potential role for anti-leukotriene therapy in pediatric OSA showed encouraging evidence that improvements in both the severity of respiratory disturbance and in the adenoid size could be anticipated in a large proportion of children with mild OSA [60]. We subsequently identified the presence of increased expression and activity of cysteinyl leukotriene receptors within lymphadenoid tissues of OSA children, and these could even be detected in exhaled condensate, serum, circulating white blood cells, or in urine [61,62,63,64,65,66,67,68,69,70]. Two RCTs have been conducted to date and have shown the favorable response to montelukast as a single agent in the treatment of pediatric OSA [71,72]. In a recent systematic review that included several different studies, significant improvements in the degree of respiratory disturbance and in the severity of hypoxemia emerged with montelukast monotherapy [73,74]. Interestingly, after a 12-week trial with either corticosteroids or oral montelukast, similar reductions in apnea-hypopnea index emerged in patients with mild OSA [75]. However, the neuropsychiatric side-effects of montelukast have possibly tilted the equipoise regarding this therapeutic option for OSA [76,77].

### 3.3. Nasal Corticosteroids and Montelukast

Based on the favorable responses to either intranasal corticosteroids or montelukast, combinatorial therapy has been proposed and thus far has resulted in rather favorable outcomes, both in terms of improvements in the severity of OSA in a large retrospective study that included 752 children [78], as well as in their quality of life [79]. In addition, combined used of nasal corticosteroids and oral montelukast appear to exhibit superior efficacy when compared to either one separately [74]. However, some children may be more likely to derive benefit from this approach than others. Indeed, obese children and children older than 7-8 years seemed less likely to exhibit favorable responses [78]. We should also emphasize that the frequency of side effects while using these agents in OSA appears to be remarkably low (0.7%) and included headaches, nausea, vomiting, and epistaxis [78].

Considering the aforementioned evidence on residual OSA, some studies have explored the potential use of anti-inflammatory therapy following T&A. Overall, the use of these agents appears to be beneficial, although the studies have been remarkably small to date and, therefore, do not specifically allow for more robust recommendations regarding their use widely [80,81,82].

## 4. Treatment of Residual OSA

In addition to anti-inflammatory therapy, non-invasive positive airway pressure (PAP) therapy has clearly been the most frequently selected treatment option for children who present with moderate to severe OSA after T&A. However, several emergent therapies have begun to be explored and merit some discussion as well (see below).

### 4.1. Weight Loss

In obese children with residual OSA, efforts to promote weight loss need to be encouraged, both as a viable therapy for OSA but also for the health promoting effects that weight loss imposes on long-term and short-term morbidity in childhood obesity [83,84,85,86]. In a multicenter study in Spain focused on obesity and OSA, the NANOS study, obese children with mild OSA with no physical evidence of adenotonsillar hypertrophy were managed with dietary interventions aimed at fostering weight loss or at least no weight gain [51]. Children who adhered to such recommendations exhibited improvements in their respiratory abnormalities with half of the sub-group displaying resolution of their OSA [50]. In another study, 61 obese adolescents underwent a multimodality inpatient treatment program consisting of moderate dietary restriction, regular physical activity, and psychological support at a residential center [85]. About 62% of this cohort had polysomnographic evidence of OSA and lost ~24kg after 4–6 months, along with major improvements in the severity of their OSA. Indeed, decreases in BMI z score were significantly associated with corresponding changes in AHI [87]. Of note, very similar findings have been recently reported out of Denmark in the context of an outpatient obesity management program [88]. A recent systematic review and meta-analysis on this issue indicates that a relatively large effect size should be expected for improvements in AHI if weight loss measures are successful in youth with OSA [89].

### 4.2. Positive Airway Pressure Therapy

As mentioned above, PAP therapy is frequently used to treat children who manifest moderate to severe OSA after T&A or as a primary intervention among those children with no evidence of enlarged tonsils and adenoids. PAP major effects are to preserve airway patency throughout the respiratory cycle while asleep, enhance functional residual capacity (FRC), and reduce the work of breathing associated with increased airway resistance. In the vast majority of patients, CPAP is implemented. However, for those patients who require very high positive end expiratory pressures, or those who suffer from medical conditions such as neuromuscular diseases or obesity hypoventilation syndrome, bilevel PAP therapy may be needed to achieve the ultimate goal of normalizing gas exchange abnormalities and enable restorative and continuous sleep throughout the night. While PAP is a highly effective therapy, achieving satisfactory adherence is a major challenge at all ages, and children are no exception. A prospective multicenter study of children randomly assigned to six months of CPAP or bilevel PAP ventilation revealed that 30% dropped out before the end of the study period, with no differences in adherence between CPAP and bilevel PAP ventilation (mean use of 5.3 ± 2.5 hours/night) [90]. Similar findings of sub-optimal adherence have been reported by others [91]. Excellent adherence is however possible, especially if PAP therapy is started in a specialized pediatric non-invasive ventilation (NIV) inpatient unit staffed by with experienced personnel and includes the use of desensitization and behavioral interventions, very frequent home visits, and repeated periodic follow-up sleep studies [92,93]. A recent retrospective analysis of more recent cohorts being treated with CPAP reveals overall good adherence, indicating that many of the educational and implementation measures are being adopted in pediatric sleep centers [94,95,96,97,98]. In addition, it appears that actually in children with intellectual disabilities, adherence which would normally be expected to be problematic can reach excellent levels [99]. Furthermore, there are clear benefits to CPAP therapy, both from a financial standpoint [100] and quality of life [101], and more importantly from a cognitive and behavioral standpoint, whereby suboptimal adherence (mean use just 170 ± 145 min/night) among 52 children resulted in significant improvements in attention span, sleepiness, internalizing and total behavior symptom scores, and caregiver and child reported quality of life [102].

Although there are clear benefits to PAP therapy, some problems and concerns deserve mention besides suboptimal adherence. Nasal bridge pressure sores from the masks, abdominal distension, oronasal dryness, eye irritation and overall discomfort from air leaks are frequent. In addition, when PAP is implemented at a very young age, flattening of the midface or maxillary retrusion from the longstanding pressure of the mask on growing facial structures may occur and needs to be carefully monitored with digital photography.

### 4.3. Drug Induced Sleep Endoscopy 

In an effort to more accurately predict the overall outcomes of T&A or to delineate an effective intervention plan among children with residual OSA, imaging techniques have emerged and are increasingly being used in clinical settings. Drug-induced sleep endoscopy (DISE) permits assessment of the upper airway using a flexible fiberoptic endoscope introduced via the nose during spontaneous breathing while the patient is under conscious sedation. The main objectives of DISE are to reconstitute the upper airway conditions as similarly as possible to those that occur during natural sleep [103]. DISE enables direct visualization of dynamic airway changes and, therefore, permits delineation of the site of obstruction and subsequent treatment planning [104]. As mentioned, DISE has rapidly gained momentum and the indications and guidelines for the use of this technique will likely emerge in the upcoming years [105,106,107,108,109,110]. At this point, it is routine clinical practice in children with residual OSA after T&A to initially offer PAP therapy and medical therapy, and when patients are either unable, unsuccessful, or unwilling to adopt such options, then use of DISE-directed surgical therapies seems a logical and reasonable step, notwithstanding its limitations [111,112]. Some authors have already advocated for use of DISE in the pre-T&A surgical planning, but results from more extensive trials are still lacking. Of note, several scoring systems exist nowadays to enable widespread standardization of DISE findings [113]. For example, the Sleep Endoscopy Rating Scale exhibits an intra-rater reliability of κ = 0.61–0.83 (substantial to excellent) and an inter-rater reliability of κ = 0.33–0.76 (fair to substantial) [114].

### 4.4. Myofunctional Therapy

Recent renewed interest in the implementation of myofunctional re-education as an approach aimed to reduce the frequency or severity of residual OSA in children. This structured intervention involves having the patients learn how to perform specific oropharyngeal exercises that target improvements in labial seal and lip tone, enhance use of nasal breathing as the preferred respiratory route, and promote more favorable positioning of the tongue within the oral cavity [115]. These focused exercises are performed daily, and they will lead to strengthening of the tongue and orofacial muscles while fostering the realignment to the correct intraoral position. To date, the studies have included only a very limited number of patients along with relatively short follow-up periods. A recent meta-analysis confirms the high level of heterogeneity of the interventions (multiple approaches and different exercises for the same objective) and, more importantly, the presence of a high risk of bias due to the low quality of the evidence [116]. Although oropharyngeal exercises have few complications and are relatively easy to teach to patients, child co-operation and adherence are essential for any potential benefit related to this type of interventions. Recent studies seem to corroborate these concerns and potentially advocate for passive myofunctional therapy via an intraoral appliance rather than the active exercise format [117,118].

### 4.5. Rapid Maxillary Expansion

Based on the assumption that increasing intraoral and upper airway introitus space will lead to reduced airflow resistance and foster airway patency, approaches aiming to achieve rapid maxillary expansion (RME) have been developed over many decades. RME usually consists of a fixed appliance with an expansion system that is affixed to opposing teeth and then progressively used to open the midpalatal suture, thus increasing the transverse diameter of the hard palate over the course of several weeks to months. The obvious theoretical advantages of such intervention are to reduce or compete eradicate residual OSA in children. However, the cumulative evidence to date on RME experience in the setting of residual OSA consists of small uncontrolled studies with a relatively short follow-up period [119,120,121,122,123,124]. Overall, it would appear that RME may have a role in carefully selected patients, more specifically in those presenting obvious malocclusion (i.e., high, narrow palate associated with deep bite, retrusive bite or crossbite) and OSA [125,126]. Younger age (during the phase of late primary dentition or early mixed dentition) is also more likely to result in favorable outcomes [125]. Future studies evaluating more critically the clinical indications and optimal ages for RME intervention, along with the potential advantage of coupling RME with T&A, are critically needed.

### 4.6. Isolated Obstructive Hypoventilation 

Obstructive alveolar hypoventilation is a term coined by Rosen and colleagues [127], and it is a frequently encountered issue in snoring children [128]. After we established the normative data for sleep studies in children regarding CO_2_ measures [129], it has become apparent that a proportion of children termed primary snorers also manifest periodic and sustained elevations of their CO_2_ levels. However, overnight end-tidal carbon dioxide levels are only weakly correlated with other polysomnographic measures. In addition, obstructive hypoventilation usually improves with T&A, but such improvements are not as predictive of the clinical response as AHI, and the degree of improvement in hypoventilation is not associated with either cognitive or behavioral changes after surgery [130]. As such, the issue of risk and benefit of treating isolated obstructive hypoventilation is completely unaddressed in the field and will have to await future studies.

## 5. Summary Conclusions

As our current understanding of the short-term and potentially long-term morbidities of pediatric OSA continues to evolve, our objectives for an increasingly effective and permanent resolution of the disease are being intensively sought. Undoubtedly, T&A remains the first line of treatment in a large majority of patients, but other approaches are insidiously entering into the fray and will progressively occupy their appropriate site in the evidence-based algorithms that need to be developed in future years. It has become apparent that no single approach will fit all patients, and that residual OSA will need to be pro-actively identified and corrected using individualized strategies that address the underlying risk factors, including better delineation of emerging therapeutic modality issues such as high flow cannula therapy, tonsillotomy versus tonsillectomy, lingual tonsil resection, and so forth. With the advent of newer imaging techniques serving as guidance before, during, and following surgical interventions, and identification of potential biomarkers serving as correlates of disease activity and morbidity, we are definitely living in exciting times and should see major breakthroughs emerge in the next several years that will lead to optimized treatment and outcomes of pediatric OSA.
